# Incidence of pulmonary vein stenosis in two types of cryoballoon systems

**DOI:** 10.1002/joa3.13087

**Published:** 2024-06-14

**Authors:** Satoko Shiomi, Michifumi Tokuda, Ryutaro Sakurai, Yoshito Yamazaki, Takuya Matsumoto, Hidenori Sato, Hirotsuna Oseto, Masaaki Yokoyama, Kenichi Tokutake, Mika Kato, Seigo Yamashita, Teiichi Yamane, Michihiro Yoshimura

**Affiliations:** ^1^ The Department of Cardiology The Jikei University School of Medicine Tokyo Japan

**Keywords:** atrial fibrillation, complication, cryoballoon, pulmonary vein isolation, stenosis

## Abstract

**Background:**

Currently, two types of cryoballoon (CB) systems are available for catheter ablation of atrial fibrillation (AF). Since the POLARx (Boston Scientific) is softer during freezing than the Arctic Front Advance Pro (AFA‐Pro; Medtronic), it tends to go more deeply into the pulmonary vein (PV), risking PV stenosis.

**Methods:**

Ninety‐one patients underwent initial CB ablation for paroxysmal AF (AFA‐Pro 56; POLARx 35). Twenty‐six from each group were extracted using propensity score matching. The PV cross‐sectional area (PVA) was measured by tracing the area within the PV plane at 5‐mm intervals from the PV ostium in a distal direction for 20 mm or to the bifurcation in each PV. The PVA was compared before and 3 months after ablation.

**Results:**

Time to balloon temperatures of −30 and − 40°C was significantly shorter and the nadir temperature was significantly lower with POLARx than with AFA‐Pro. In the left inferior (LI) PV and right superior (RS) PV, the freezing balloon position was significantly deeper in POLARx than in AFA‐pro. The freezing position in RSPV with mild to moderate narrowing was deeper than those without (10.2 ± 3.3 mm vs. 8.2 ± 1.8 mm, *p* = .01). In RSPV, the reduction of PVA tended to be greater with the POLARx than with the AFA‐Pro (26.1% ± 14.1% vs. 19.9% ± 10.3%, *p* = .07).

**Conclusion:**

There was no significant difference in the incidence of PV stenosis between POLARx and AFA‐Pro. However, if POLARx goes deep into the PVs, we will still have to be careful.

## Graphical abstract

There was no significant difference in the incidence of PV stenosis between POLARx and AFA‐Pro. However, POLARx goes deep into the PVs, we will still have to be careful.

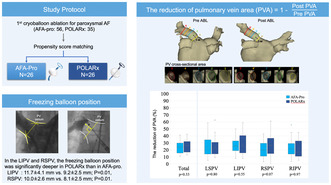

## 1 INTRODUCTION

Atrial fibrillation (AF) is an arrhythmia that induces heart failure and cerebral infarction, and catheter ablation has gained attention as a radical treatment. The most crucial aspect of AF ablation is to isolate the pulmonary veins (PVs). A cryoballoon (CB) is a device that completes PV isolation in a single shot by freezing the PV ostium. However, PV stenosis sometimes occurs after PV isolation and remains a major complication requiring intervention, associated with significant morbidity.^1^, ^2^, ^3^ Having a large PV diameter at the baseline, freezing taking a long time, and a low nadir temperature was determined to be risk factors for PV stenosis.^1^, ^2^, ^4^

Two types of CB systems are currently available for pulmonary vein isolation (PVI). Since the POLARx (Boston Scientific, Natick, MA, USA) is softer during freezing than the Arctic Front Advance Pro (AFA‐Pro; Medtronic, Minneapolis, MN, USA), it tends to go more deeply into the PV, risking PV stenosis. PV stenosis may thus occur more frequently when using the POLARx than when using the AFA‐pro. To clarify this point, we compared the PV area reduction and incidence of PV stenosis after catheter ablation of AF using the AFA‐pro and POLARx. The PV area was measured using the 3D electro‐anatomical mapping system by tracing the area within the PV plane. PV ostium was defined as the intersection of 2 straight lines, which were drawn along the outlines of the left atrium (LA) and PV.

## 2 METHODS

### 2.1 Study population

Ninety‐one patients underwent initial CB ablation for paroxysmal AF (AFA‐Pro in 56; POLARx in 35) between January 2021 and December 2022. POLARx began use in April 2021. The specific balloon used for each participant was left to the operator's discretion. We did not use a CB for patients with PV anomalies, such as common PV and middle PV. Paroxysmal AF was defined as AF that terminated spontaneously within 7 days. All patients received effective anticoagulation therapy for over 3 weeks before the procedure. All antiarrhythmic drugs were discontinued at least five half‐lives before the procedure.

This study was approved by the Ethics Committee of The Jikei University School of Medicine for Biomedical Research (study protocol: 29–361(8977)). We complied with the routine ethical regulations of our institution. All clinical investigations were conducted according to the principles outlined in the Declaration of Helsinki. We also posted a notice about the study design and contact information on our institution's website. As this was a retrospective study, the requirement for informed consent was waived by the Ethics Committee of The Jikei University School of Medicine for Biomedical Research.

Propensity matching was performed to minimize the effect of potential confounders from selection bias. After the propensity score matching analysis, 26 patients each who underwent procedures using these CB devices remained.

### 2.2 Electrophysiological study and AF ablation

The details of AF ablation using CB have been reported previously.^3^, ^4^ All procedures were performed under deep sedation with propofol. A 15‐Fr (Flexcath Advance; Medtronic) or 15.9‐Fr steerable sheath (POLARSHEATH; Boston Scientific) was introduced into the LA. An inner lumen mapping catheter (Achieve; Medtronic or POLARMAP; Boston Scientific) was sequentially positioned in each PV to obtain the baseline PV potential. A 28‐mm CB (AFA‐Pro or POLARx) was advanced into the LA over the inner lumen mapping catheter, inflated, and positioned in the PV antrum of each vein. The CB was inflated and advanced to the ostium of each PV to obtain complete occlusion. The degree of occlusion was assessed based on the grade of retention of the contrast medium injected into the PV through the inner lumen of the CB.

After total occlusion was thought to have been achieved with complete contrast retention, the CB was withdrawn slightly, and a leak around the PV balloon interface was allowed to better define the PV ostium and ensure proximal ablation (proximal seal technique) to occlude the PV as proximally as possible at the ostium. Freezing was usually started only if complete occlusion had been achieved. The disappearance of PV potential is used as an indicator of PV isolation, and if isolation cannot be achieved, a touch‐up with radiofrequency is added.

To avoid esophageal injury, a nasogastric thermometer (Sensi Therm; St. Jude Medical, St. Paul, MN, USA) was inserted to identify the course of the esophagus and measure the esophageal temperature during CB ablation. After verifying the complete occlusion of PV ostium, cryo energy was applied for 180 seconds. CB applications for right PVs were performed to monitor the diaphragmatic compound motor action potentials (CMAP) during phrenic nerve pacing to avoid phrenic nerve injury. In cases with either a minimum temperature of <−60°C with the AFA‐Pro or <−70°C with the POLARx, an esophageal temperature of <15°C, a CMAP amplitude drop >30% from baseline, or in which the PV potential remained after a 100‐s application of cryo‐energy, CB ablation was stopped. If the PV potential did not disappear after the second application at the same PV, touch‐up ablation with an irrigated ablation catheter was added. After CB ablation of all four PVs, a bidirectional block at the PV antrum was confirmed using a variable circular mapping catheter (Lasso 2515 NAV eco variable catheter; Biosense Webster, Diamond Bar, CA, USA).

### 2.3 The evaluation of the PV morphology

The PV cross‐sectional area (PVA) was compared between contrast‐enhanced multidetector computed tomography (Somatom Drive, Siemens Medical Solutions, Forchheim, Germany) before and 3 months after ablation. Three‐dimensional (3D) images of the LA and PVs were reconstructed from multidetector computed tomography images using a 3D electroanatomical mapping system (Ensite NavX; St. Jude Medical). Two independent experts (H.S. and H.O.) measured PV cross‐sectional area, and balloon depth and angle were measured by a single person (H.O.) blinded to the patient's background and procedural results. Both measurements were performed by persons other than the operator. The PVA was calculated using the 3D electro‐anatomical mapping system by tracing the area within the PV plane at 5‐mm intervals from the PV ostium in a distal direction for 20 mm or to the bifurcation in each PV, as previously described.^4^ The PVA reduction rate was calculated at each segment using the following formula: [1 – (PVA_post_/PVA_pre_)] × 100 (%). The ΔPVA was defined as the maximum PVA reduction rate in each PV.^4^ PV stenosis was classified into three groups according to the ΔPVA value: mild narrowing (25%–50%), moderate stenosis (50%–75%), and severe stenosis (>75%). The average PVA was defined as the average area measured every 5 mm for each PV. An excellent interobserver correlation was observed in measuring PV cross‐sectional area (kappa coefficient: 0.89).

### 2.4 Balloon position depth

**FIGURE 1:**
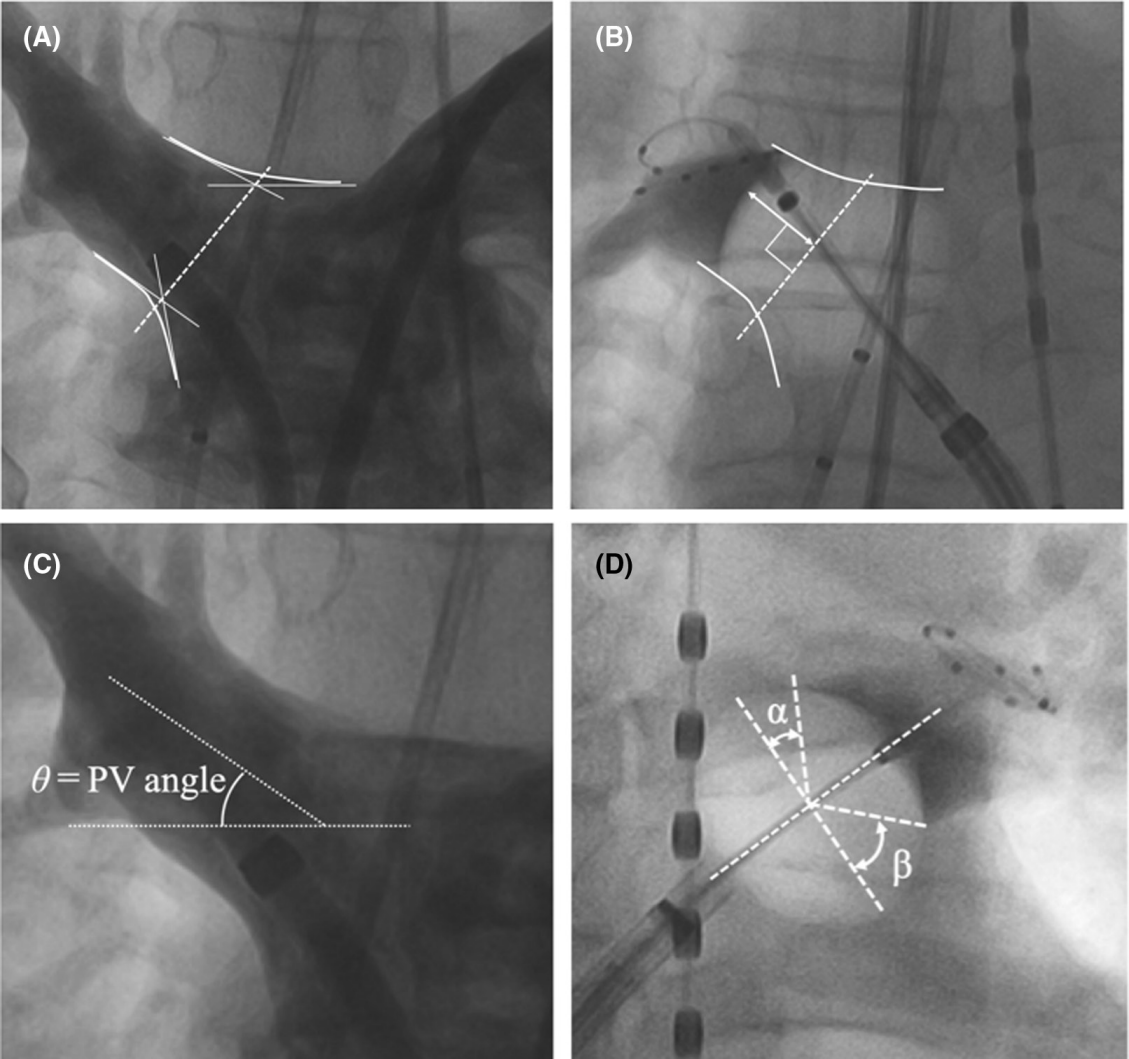
(A, B) Depth of the balloon position on angiography, defined as the length from the PV ostial level of the balloon vertically. (C) The pulmonary vein angle on angiography was defined as the internal angle between each PV and a horizontal line. (D) The contact angle between the cryoballoon and pulmonary vein wall on angiography. Balloon contact ratio; (*α* + *β*)/180. PV, pulmonary vein.

The depth of the balloon position was evaluated as previously reported.^3^ We assessed the localization of the balloon position during PV occlusion in each PV. The PV ostium (ostial level) on angiography was defined as the intersection of two straight lines drawn along the outlines of the LA and PV (Figure 1A). The depth of the balloon position was determined and measured as the length between the arctic apex of the balloon and the PV ostium on angiography at first application (Figure 1B). The pulmonary vein angle on angiography was defined as the internal angle between each PV and a horizontal line (Figure 1C). We measured the balloon‐contacting angles from the equator line (roof side: *α*, bottom side: *β*). The balloon contact ratio (BCR) was defined as (*α* + *β*)/180 (Figure 1D).^3^

### 2.5 Statistical analyses

Continuous variables were expressed as the mean ± standard deviation (SD). An unpaired Student's *t*‐test or the Mann–Whitney U test was used for continuous variables. Categorical variables, defined as numbers or proportions, were analyzed using the chi‐square test unless the expected values in any cells were <5, in which Fisher's exact test was used. All *p*‐values of <.05 were considered to indicate statistical significance. The Pearson correlation coefficient (*r*) was used to examine the relationship between the two groups.

Propensity scores were calculated for each of the 91 patients based on a multivariable logistic regression model. A total of 16 characteristics (all variables in Table 1) hypothesized to be associated with the outcomes of catheter ablation were assessed for inclusion in the model as independent variables. All 16 characteristics were retained in the model with stepwise selection and were subsequently used to generate propensity scores. In the selection process, a *p* value of .05 was used as a cutoff for a characteristic to be entered and remained in the model. In order of stepwise selection, the matching variables were as follows: gender, age, body mass index (BMI), LA diameter (LAD), left ventricular ejection fraction (LVEF), estimated glomerular filtration rate (eGFR), B‐type natriuretic peptide (BNP), CHA_2_DS_2_‐VASC score, hypertension, diabetes mellitus, congestive infarction, vascular disease, and original PVA. The baseline characteristics after matching were assessed by evaluating the standardized mean differences; a standardized mean difference numerically who underwent PVI with AFA‐Pro and POLARx were matched on a 1:1 basis with the nearest neighbor algorithm without replacement (caliper width: 1/5 logit of the standard deviation). All statistical analyses were performed using the SPSS software program (version 28.0.0.0.; SPSS, Chicago, IL, USA).

**TABLE 1:** Patient background characteristics before and after propensity score matching.

	All			Post propensity score matching		
	AFA‐pro (*n* = 56)	POLARx (*n* = 35)	*p*	AFA‐pro (*n* = 26)	POLARx (*n* = 26)	*p*
Gender (female)	9 (16%)	11 (31%)	0.11	6 (23%)	7 (27%)	0.75
Age (y/o)	63.4 ± 11.6	59.7 ± 11.3	0.14	62.4 ± 12.7	60.8 ± 10.0	0.60
BMI	24.7 ± 3.4	23.4 ± 3.9	0.10	23.4 ± 3.0	23.4 ± 3.7	0.98
LAD (mm)	37.0 ± 6.0	34.0 ± 5.8	0.02	34.6 ± 6.0	34.4 ± 5.2	0.89
LVEF (%)	62.8 ± 8.2	64.4 ± 5.5	0.31	64.2 ± 6.3	64.0 ± 5.9	0.91
eGFR (mL/min/1.73 m^2^)	72.4 ± 14.6	73.4 ± 14.4	0.74	72.2 ± 13.2	73.9 ± 15.0	0.66
BNP	38.2 ± 35.0	42.4 ± 47.7	0.63	37.3 ± 35.1	35.3 ± 27.8	0.82
Risk score						
CHA_2_DS_2_‐VASC score	1.5 ± 1.1	1.6 ± 1.2	0.66	1.7 ± 1.1	1.6 ± 1.2	0.64
Comorbidities (%)						
Hypertension	25 (45%)	20 (57%)	0.25	14 (54%)	14 (54%)	1.00
Diabetes	8 (14%)	3 (8.6%)	0.42	2 (7.7%)	3 (12%)	0.65
Cerebral infarction	1 (1.8%)	3 (8.6%)	0.19	1 (3.8%)	2 (7.7%)	0.56
Vascular disease	1 (1.8%)	0 (0%)	0.43	1 (3.8%)	0 (0%)	0.33
Original PVA (cm^2^)						
LSPV	3.0 ± 0.6	3.1 ± 0.6	0.27	3.2 ± 0.6	3.0 ± 0.5	0.32
LIPV	2.4 ± 0.5	2.5 ± 0.3	0.31	2.6 ± 0.5	2.5 ± 0.3	0.78
RSPV	3.1 ± 0.7	3.0 ± 0.5	0.49	3.2 ± 0.9	3.1 ± 0.5	0.55
RIPV	2.6 ± 0.5	2.6 ± 0.3	0.85	2.6 ± 0.5	2.6 ± 0.4	0.85

*Notes*: The data are presented as the mean ± standard deviation, median (interquartile range), or *n* (%). The original PVA was compared at the PV ostium.

Abbreviations: BMI, body mass index; BNP, brain natriuretic peptide; eGFR, estimated glomerular filtration rate; LAD, left atrium diameter; LI, left inferior; LS, left superior; LVEF, left ventricle ejection fraction; PVA, pulmonary vein area; RI, right inferior; RS, right superior.

## 3 RESULTS

### 3.1 Patient characteristics

Ninety‐one patients (AFA‐Pro [Group A] in 56 and POLARx [Group P] in 35) were enrolled in this study. Patient characteristics are shown in Table 1. Propensity score matching was performed to minimize differences between the groups. Before propensity score matching, the LAD was larger in Group A than in Group P. Fifty‐two patients (Group A: 26, Group P: 26) were selected by propensity score matching. After the propensity score was matched, the patient characteristics were similar between the two groups.

### 3.2 Ablation results

Procedural parameters between the two groups are shown in Table 2. In the left inferior (LI) PV and right superior (RS) PV, the freezing balloon position was significantly deeper in Group P than in Group A (LIPV: 11.7 ± 4.1 mm vs. 9.2 ± 2.5 mm; *p* = .01, RSPV: 10.0 ± 2.6 mm vs. 8.1 ± 2.5 mm; *p* = .01). For the RSPV, significantly more CB applications were required in Group P than in Group A. When compared to the mild PV narrowing (25%–50%) (35 PVs in Group A and 44 PVs in Group P) and moderate (50%–75%) PV stenosis (4 PVs in each group), Group P has a deeper balloon position than Group A (11.9 ± 3.6 mm vs. 9.5 ± 2.6 mm; *p* = .001). There was a positive correlation between the number of approaches to the RSPV and the balloon depth (*r* = .045). Radiofrequency touch‐up ablation was required at similar rates between the two groups. Cavo‐tricuspid isthmus line ablation tended to be more frequently performed, and the total procedure time was longer in Group A than in Group P. The incidence of CMAP reduction (>30%) was similar between Group P and Group A (12% vs. 3.9% *p* = .31). All cases of CMAP reduction occurred during application at RSPV. Transient phrenic nerve injury occurred in one case in each group.

**TABLE 2:** The comparison of the AFA‐pro and POLARx.

	Group A (*n* = 26)	Group P (*n* = 26)	*p*
PV angle (°)			
LSPV	27.8 ± 10.9	29.5 ± 13.3	0.61
LIPV	11.1 ± 5.6	15.9 ± 9.4	0.03
RSPV	37.9 ± 8.4	42.6 ± 8.8	0.05
RIPV	13.4 ± 8.2	14.9 ± 8.9	0.55
Depth of balloon position (mm)			
LSPV	9.6 ± 2.9	11.1 ± 2.8	0.07
LIPV	9.2 ± 2.5	11.7 ± 4.1	0.01
RSPV	8.1 ± 2.5	10.0 ± 2.6	0.01
RIPV	10.2 ± 3.5	10.6 ± 2.7	0.64
Balloon contact ratio			
LSPV	0.44 ± 0.1	0.49 ± 0.1	0.05
LIPV	0.52 ± 0.1	0.57 ± 0.1	0.03
RSPV	0.47 ± 0.1	0.50 ± 0.1	0.28
RIPV	0.59 ± 0.1	0.57 ± 0.1	0.51
Number of CB application (*n*)			
LSPV	1.3 ± 0.5	1.2 ± 0.4	0.40
LIPV	1.2 ± 0.4	1.1 ± 0.3	0.23
RSPV	1.0 ± 0.2	1.4 ± 0.7	0.02
RIPV	1.1 ± 0.3	1.2 ± 0.4	0.21
Touch up application (%)			
LSPV	1 (3.8%)	1 (3.8%)	1.00
LIPV	1 (3.8%)	1 (3.8%)	0.96
RSPV	0	0	1
RIPV	8 (31%)	3 (12%)	0.12
Total procedure time (min)	167 ± 49	137 ± 42	0.03
SVCI (%)	3 (12%)	2 (7.7%)	0.65
CTI ablation (%)	15 (58%)	8 (31%)	0.05
Non‐PV (%)	2 (7.7%)	2 (7.7%)	1
CMAP reduction (%)	1 (3.8%)	3 (12%)	0.31
PNI (%)	1 (3.8%)	1 (3.8%)	1

*Note*: The data are presented as the mean ± standard deviation, median (interquartile range), or *n* (%).

Abbreviations: CB, cryoballoon; CMAP, compound motor action potential; CTI, cavo tricuspid isthmus; PNI, phrenic nerve injury; PV, pulmonary vein; SVCI, superior vena cava isolation.

The procedural parameters are shown in Table 3. The time to −30 or −40°C was shorter in Group P than in Group A (*p* < .001, except for time to −30°C at the RSPV). While the nadir temperature was lower in Group P than in Group A (*p* < .001) in all PVs, the time to isolation was similar between the two groups. The thawing time to 0°C was longer in Group P than in Group A.

**TABLE 3:** Freezing parameters and pulmonary vein stenosis rate before and after ablation.

	Group A (*n* = 26)	Group P (*n* = 26)	*p*
LSPV			
Time to balloon temperature − 30°C (s)	31.5 ± 6.4	26.2 ± 1.8	<0.001
Time to balloon temperature − 40°C (s)	60.6 ± 24.0	29.7 ± 2.5	<0.001
Nadir temperature (°C)	−50.4 ± 4.9	−61.2 ± 3.8	<0.001
Time to isolation (s)	44.7 ± 18.3	40.3 ± 21.5	0.45
Thawing time to 0°C (s)	32.0 ± 9.7	58.0 ± 18.7	<0.001
ΔPVA (%)	23.8 ± 13.7	24.2 ± 12/4	0.91
25% ≦ΔPVA <50% (%)	7 (27%)	9 (35%)	0.56
50% ≦ΔPVA <75% (%)	2 (7.7%)	1 (3.8%)	0.56
LIPV			
Time to balloon temperature − 30°C (s)	31.5 ± 6.4	26.2 ± 1.8	<0.001
Time to balloon temperature − 40°C (s)	60.6 ± 24.0	29.7 ± 2.5	<0.001
Nadir temperature (°C)	−50.4 ± 4.9	−61.2 ± 3.8	<0.001
Time to isolation (s)	44.7 ± 18.3	40.3 ± 21.5	0.45
Thawing time to 0°C (s)	51.4 ± 13.2	79.0 ± 29.3	<0.001
ΔPVA (%)	25.7 ± 11.1	27.8 ± 14.5	0.55
25% ≦ΔPVA <50% (%)	13 (50%)	12 (46%)	0.79
50% ≦ΔPVA <75% (%)	1 (3.8%)	2 (7.7%)	0.56
RSPV			
Time to balloon temperature − 30°C (s)	25.7 ± 4.0	24.2 ± 3.4	0.15
Time to balloon temperature − 40°C (s)	42.2 ± 9.5	27.5 ± 3.9	<0.001
Nadir temperature (°C)	−54.0 ± 5.0	−65.0 ± 5.6	<0.001
Time to isolation (s)	24.4 ± 12.2	29.0 ± 14.0	0.23
Thawing time to 0°C (s)	53.2 ± 13.1	78.8 ± 33.8	0.01
ΔPVA (%)	19.9 ± 10.3	26.1 ± 14.1	0.07
25% ≦ΔPVA <50% (%)	7 (27%)	14 (54%)	0.049
50% ≦ΔPVA <75% (%)	0 (0%)	1 (3.8%)	0.33
RIPV			
Time to balloon temperature − 30°C (s)	31.6 ± 8.6	25.1 ± 1.7	<0.001
Time to balloon temperature − 40°C (s)	61.1 ± 23.8	28.8 ± 3.2	<0.001
Nadir temperature (°C)	−48.3 ± 6.8	−59.6 ± 6.5	<0.001
Time to isolation (s)	36.6 ± 22.4	42.4 ± 33.6	0.61
Thawing time to 0°C (s)	35.8 ± 13.6	65.2 ± 23.3	<0.001
ΔPVA (%)	21.8 ± 11.8	21.9 ± 12.1	0.97
25% ≦ΔPVA <50% (%)	8 (31%)	9 (35%)	0.77
50% ≦ΔPVA <75% (%)	1 (3.8%)	0 (0%)	0.33

*Notes*: The data are presented as the mean ± standard deviation or median (interquartile range).

The data are presented as the mean ± standard deviation, median (interquartile range), or n (%).

Abbreviations: LI, left inferior; LS, left superior; PV, pulmonary vein; RI, right inferior; RS, right superior; ΔPVA, [1 – (PVA_post_/PVA_pre_)] × 100 (%).

### 3.3 PV area reduction following CB ablation

**FIGURE 2:**
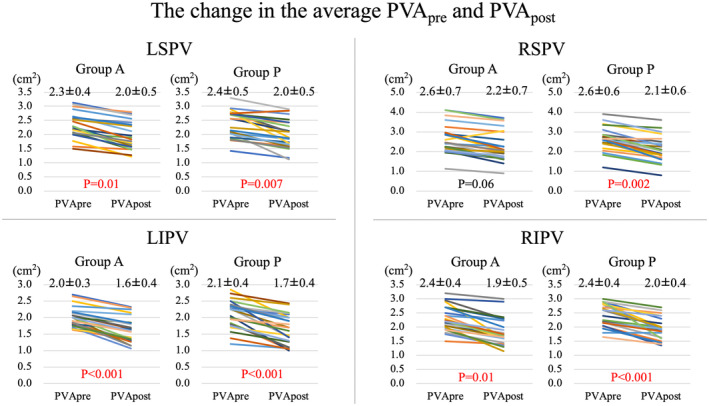
Changes in the average of all PVAs after ablation. Each change for Groups A and P is shown. PVA, PV cross‐sectional area.

The average PV cross‐sectional area pre‐ablation (PVA_pre_) is shown in Table 1, and the change in the average PVA (ΔPVA) is in Figure 2. The average PVA_pre_ and PVA_post_ were similar between the two groups. In Group A, the average PVA_post_ of the LSPV, LIPV, and RIPV were significantly reduced compared with the average PVA_pre_. In Group P, the average PVA_post_ of all PVs was significantly smaller than the average PVA_pre_ (Figure 2).

**FIGURE 3:**
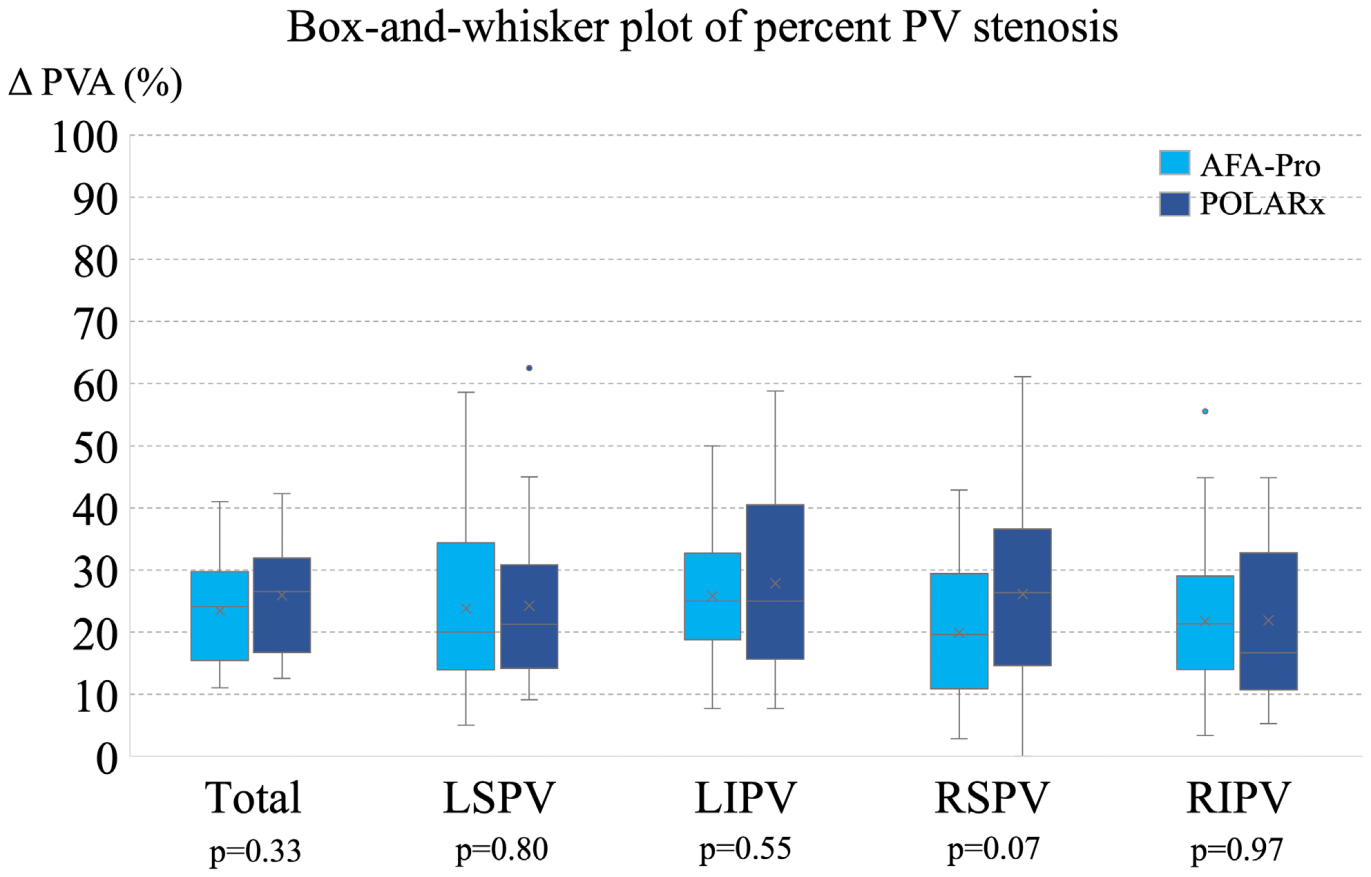
Box‐and‐whisker plot of the percentage of PV stenosis. Each graph shows the AFA‐Pro on the left and the POLARx on the right. PV, pulmonary vein.

In all four PVs, the ΔPVA was not significantly different between Group P and A. However, in the RSPV, the reduction in the PVA tended to be larger in Group P than in Group A (26.1% ± 14.1% vs. 19.9% ± 10.3%, *p* = .07, Figure 3). Mild PV narrowing (25%–50%) at RSPV more frequently occurred in Group P than in Group A (54% vs. 27%; *p* = .049). Moderate PV stenosis (50%–75%) was observed in 8 PVs in 8 patients (2 LSPVs, 1 LIPV, and 1 RIPV in Group A and 1 LSPV, 2 LIPVs, and 1 RSPV in Group P). There was no significant difference in the incidence of moderate PV stenosis between the two groups (Table 3). There was no severe stenosis in either group. In RSPVs with mild to moderate narrowing, the freezing balloon position was deeper than those without (10.2 ± 3.3 mm vs. 8.2 ± 1.8 mm, *p* = .01).

**FIGURE 4:**
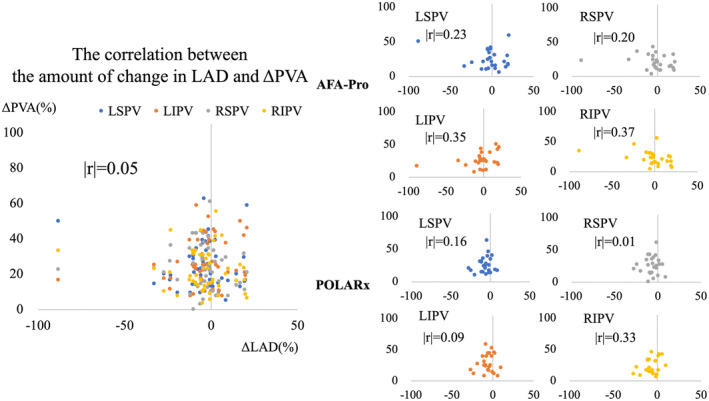
The correlation between the change in the LAD and PVA is |*r*| = 0.05; no correlation was noted even when comparing each PV of each group. LAD, left atrial dimension; PV, pulmonary vein; PVA, PV cross‐sectional area.

The correlation between the reduction in the LAD and ΔPVA was investigated regarding the effect of negative remodeling of the LA and PV. The result showed no correlation between the decrease in the LAD and ΔPVA (|*r*| = .05) (Figure 4).

## 4 DISCUSSION

### 4.1 Main findings

With the POLARx, the time to −30 or − 40°C was shorter, the nadir temperature was lower, and the thawing time to 0°C was longer than with an AFA‐Pro. Still, there was no significant difference in the isolation time between the devices. In the LIPV and RSPV, the balloon went significantly deeper into the PV in Group P than Group A. On comparing the PVA before and after the ablation, the PVA reduction in the RSPV tended to be greater with the POLARx than with the AFA‐Pro. In RSPVs with mild to moderate narrowing, the freezing balloon position was deeper than those without.

### 4.2 Mechanism underlying PV stenosis and therapy effects

PV stenosis caused by a CB is observed in 2%–3% of cases, and severe stenosis has also been reported.^1^, ^2^, ^3^ The mechanism underlying PV stenosis after radiofrequency ablation was considered to involve a scarring response to thermal injury.^5^

In contrast to radiofrequency, which leads to diffuse cell destruction and denaturation of the extracellular tissue matrix, the mechanism of stenosis with CB involves extracellular ice formation during freezing, increasing the extracellular osmotic pressure, resulting in cell dehydration and cell shrinkage^6^ CB ablation causes minimum damage to connective tissue^7^ and pathologically only mild fibrosis of the intima,^8^ with minimal tissue contraction observed upon healing of the lesion and a theoretically reduced incidence of venous stenosis. However, the incidence of severe PV stenosis after CB ablation is comparable to that after radiofrequency ablation.^9^ Several risk factors of PV stenosis after CB ablation were previously reported. PV stenosis has been reported to be favorably associated with a large‐diameter PV at the baseline.^4^ Furthermore, a longer time to freeze and lower nadir temperature were found to lead to PV stenosis.^1^, ^2^ Freezing inside the PV, which has a large contact area with the CB and lower balloon temperature, was considered to carry a risk of PV stenosis.

### 4.3 The AFA‐pro versus POLARx

Two types of balloons are currently used, both of which have a size of 28 mm, and are mainly used in clinical practice. All PVs were thus isolated with a 28‐mm CB in this study. However, the AFA‐Pro does not reach 28 mm before freezing starts,^10^ and when cooling does start, the cooling flow rate rises rapidly along with the pressure.^11^ The POLARx uses a proprietary thermoplastic material and is a soft balloon. Since it is equipped with a pressure sensor, the pressure is constant after inflation, making it difficult to deform.^12^ Because the POLARx is soft, it has the advantage of not pushing back when freezing. However, this makes it easier for the POLARx to go deep into the PV. Thus, there may be a risk of PV stenosis due to freezing at a deeper position with this device. However, no study has compared the reduction of the PV area between the AFA‐pro and POLARx.

We hypothesized that PV stenosis may occur more frequently with the POLARx than with the AFA‐pro. Similar to previous studies, the POLARx reached temperatures of −30 and − 40°C earlier than the AFA‐pro, its nadir temperature was lower, and its thawing time to 0°C was also longer. However, there was no significant difference in the time to isolation between the two groups. The balloon position was deeper with the POLARx than with the AFA‐Pro in the LIPV and RSPV, and the reduction in the PVA tended to be larger in the RSPV with the POLARx. The freezing balloon position was deeper in RSPV with mild to moderate PV narrowing than those without. A previous report found that the POLARx was better at pressing to the RPV than the AFA‐Pro.^13^ Good contact in that case was thought to be due to good stability thanks to its deep position in the PV, which was consistent with the results of this study. Deep insertion of the CB into the PV was considered a cause of PV stenosis, and it was thought that the POLARx should be gently pressed to the PV ostium without pushing it into the PVs due to concerns about PV stenosis. Freezing at too shallow position to prevent stenosis will conversely cause poor occlusion, increase the number of CB applications, and force the operator to choose freezing at a deeper position. Care should be taken when using these devices, as the POLARx is more flexible than the AFA‐Pro and can thus go more deeply into the PV. This point may be overcome using a new model (POLARx FIT) that allows further enlargement to 31 mm.

### 4.4 Study limitations

Several limitations associated with the present study warrant mention. This was a retrospective, single‐center, and non‐randomized study. In addition, it was performed by multiple operators, and there was a possibility that the procedures needed to be unified. The specific balloon used for each participant was left to the operator's discretion, which may have introduced selection bias. Although the pre‐procedural PV size was similar between the two groups, preoperative computed tomography results may have influenced device selection. In addition, since fluoroscopy was two‐dimensional, the balloon contact and angle could not be measured accurately in some cases. The small number of patients may have been the reason for the lack of a significant difference in the rates of PV stenosis.

## 5 CONCLUSION

There was no significant difference in the incidence of PV stenosis between POLARx and AFA‐Pro. However, if POLARx goes deep into the PVs, we will still have to be careful.

## FUNDING INFORMATION

None.

## CONFLICT OF INTEREST STATEMENT

Michifumi Tokuda received a consulting fee from Medtronic and research funding from Japan Lifeline Co. LTD, and Teiichi Yamane received speaker honoraria from Medtronic Japan and research grants from Japan Lifeline Co. LTD. Other authors declare that they have no conflicts of interest.

## ETHICS APPROVAL STATEMENT

Clinical investigations were conducted according to the principles expressed in the Declaration of Helsinki.

## APPROVAL OF THE RESEARCH PROTOCOL

This study was approved by the Ethics Committee of The Jikei University School of Medicine for Biomedical Research (study protocol: 29–361(8977)).

## PERMISSION TO REPRODUCE MATERIAL FROM OTHER SOURCES

None

## REGISTRY AND THE REGISTRATION NO. OF THE STUDY/TRIAL

N/A.

## ANIMAL STUDIES

N/A.

## Data Availability

Data sharing is not applicable to this article as no datasets were generated or analyzed during the current study.

## References

[1] Miyazaki S , Kajiyama T , Hada M , Nakamura H , Hachiya H , Tada H , et al. Does second‐generation cryoballoon ablation using the current single short freeze strategy produce pulmonary vein stenosis? Int J Cardiol. 2018;272:175–178. 10.1016/j.ijcard.2018.08.004 30093139

[2] Matsuda J , Miyazaki S , Nakamura H , Taniguchi H , Kajiyama T , Hachiya H , et al. Pulmonary vein stenosis after second‐generation Cryoballoon ablation. J Cardiovasc Electrophysiol. 2017;28:298–303. 10.1111/jce.13155 28032927

[3] Tokutake K , Tokuda M , Yamashita S , Sato H , Ikewaki H , Okajima E , et al. Anatomical and procedural factors of severe pulmonary vein stenosis after Cryoballoon pulmonary vein ablation. JACC Clin Electrophysiol. 2019;5:1303–1315. 10.1016/j.jacep.2019.08.003 31753437

[4] Narui R , Tokuda M , Matsushima M , Isogai R , Tokutake K , Yokoyama K , et al. Incidence and factors associated with the occurrence of pulmonary vein narrowing after Cryoballoon ablation. Circ Arrhythm Electrophysiol. 2017;10:10. 10.1161/CIRCEP.116.004588 28630168

[5] Taylor GW , Kay GN , Zheng X , Bishop S , Ideker RE . Pathological effects of extensive radiofrequency energy applications in the pulmonary veins in dogs. Circulation. 2000;101:1736–1742. 10.1161/01.cir.101.14.1736 10758058

[6] Erinjeri JP , Clark TW . Cryoablation: mechanism of action and devices. J Vasc Interv Radiol. 2010;21:S187–S191. 10.1016/j.jvir.2009.12.403 20656228 PMC6661161

[7] Shepherd JP , Dawber RP . Wound healing and scarring after cryosurgery. Cryobiology. 1984;21:157–169. 10.1016/0011-2240(84)90207-4 6713945

[8] Feld GK , Yao B , Reu G , Kudaravalli R . Acute and chronic effects of cryoablation of the pulmonary veins in the dog as a potential treatment for focal atrial fibrillation. J Interv Card Electrophysiol. 2003;8:135–140. 10.1023/a:1023660901347 12766505

[9] Tokuda M , Yamashita S , Shiomi S , Sakurai R , Sato H , Oseto H , et al. Pulmonary vein stenosis after catheter ablation of atrial fibrillation using a Cryoballoon, hot balloon, or laser balloon. Circ J. 2023;87:1711–1719. 10.1253/circj.CJ-23-0048 37258224

[10] Yap SC , Anic A , Breskovic T , Haas A , Bhagwandien RE , Jurisic Z , et al. Comparison of procedural efficacy and biophysical parameters between two competing cryoballoon technologies for pulmonary vein isolation: insights from an initial multicenter experience. J Cardiovasc Electrophysiol. 2021;32:580–587. 10.1111/jce.14915 33492749 PMC7986676

[11] Andrade JG . Cryoablation for atrial fibrillation. Heart Rhythm. 2020;O2(1):44–58. 10.1016/j.hroo.2020.02.004 PMC8183843 34113858

[12] Tomaiko‐Clark E , Bai R , Khokhar M , Su WW . A tale of two balloons: technical and procedural difference between cryoballoon systems. Curr Opin Cardiol. 2022;37:62–67. 10.1097/HCO.0000000000000942 34783712 PMC8711604

[13] Creta A , Kanthasamy V , Schilling RJ , Rosengarten J , Khan F , Honarbakhsh S , et al. First experience of POLARx versus Arctic front advance: an early technology comparison. J Cardiovasc Electrophysiol. 2021;32:925–930. 10.1111/jce.14951 33590568

